# What's in a name: The impact of monkeypox nomenclature

**DOI:** 10.1002/puh2.25

**Published:** 2022-11-02

**Authors:** Lawson Ifeanyi Eya, Muiz Ibrahim, Creuza Rachel Vicente, Adrian Paul J. Rabe

**Affiliations:** ^1^ Noonu Atoll Hospital Noonu Atoll Maldives; ^2^ Global Health Focus Abuja Nigeria; ^3^ International Higher School of Medicine International University of Kyrgyzstan Bishkek City Kyrgyzstan; ^4^ Department of Social Medicine Federal University of Espírito Santo Vitória Brazil; ^5^ Postgraduate Program in Infectious Diseases (PPGDI) Federal University of Espírito Santo Vitória Brazil; ^6^ Faculty of Medicine Department of Primary Care and Public Health School of Public Health Imperial College London London UK

**Keywords:** global health, monkeypox, pandemic, viral nomenclature

## Abstract

The monkeypox outbreak has garnered significant global attention and has since been declared a Public Health Emergency of International Concern (PHEIC). Even though the virus is not novel and has been endemic in African countries, most cases in the present outbreak have been detected in Europe and the Americas. Disease nomenclature has historically included references to places, animals, and people. There could be unintended negative consequences emanating from these references. The COVID‐19 pandemic has exposed some of these consequences, with terms such as “China virus” and fueling racism and hate crimes toward Asians, especially in America. Similarly, following the monkeypox outbreak, there have been reports of attacks on monkeys in Brazil even though monkeys are not linked to this present outbreak. The WHO Best Practices for the Naming of New Human Infectious Diseases has advised the avoidance of these references and terms that can cause negative impacts. The naming of the COVID‐19 virus is an example of the use of the new naming practice. The WHO is already working on new names for the monkeypox disease. This name change will help clear up the confusion and prevent further attacks on monkeys both in Brazil and other parts of the world.

## INTRODUCTION

While the world recovers from the massive global disruption caused by the COVID‐19 virus (SARS‐CoV‐2), a new virus is already spreading around the world. Unlike COVID‐19, the monkeypox disease is not novel. Monkeypox is a zoonotic infection originating from the monkeypox virus, a DNA virus of the Poxviridae family and the Orthopoxvirus genus which is closely related to the smallpox virus [1]. The virus was first identified in monkeys in a laboratory in Denmark in 1958, garnering the name “monkeypox” [2]. It was not until 1970 that the first human case was reported in a child in the Democratic Republic of the Congo [2]. Monkeypox has been endemic in Africa where sporadic outbreaks have occurred [1]. Other non‐endemic countries such as the United States, the United Kingdom, Israel, and Singapore have reported a few cases of imported monkeypox infections in the past [1].

In this recent global outbreak of monkeypox, most of the reported cases are in European and American regions [3]. So far, 27,814 laboratory‐confirmed cases and 11 deaths from 89 countries were reported to the WHO between 1 January and 7 August 2022 [3]. This is the first period where deaths attributed to monkeypox have been reported outside the WHO African Region [3]. The increasing number of reported cases poses a global public health threat, hence the WHO declared the global monkeypox outbreak a Public Health Emergency of International Concern (PHEIC) on the 23 July 2022 [4].

Historically, disease names have included references to geographical locations, animals, and even people [5]. These names were originally not intended to cause any harm; some naming conventions allow attribution to the individuals or places related to discovery, according to an element of prestige and recognition. Some of the disease nomenclatures based on association with an animal or a place could be misnomers because identifying a virus from a particular geographic location, does not necessarily mean that it originated from there [6]. Instances of misnomers include the “Spanish flu” which may have originated in the United States before spreading to Europe, and “Marburg disease” which did not originate in Germany [6]. There are also some diseases like monkeypox which can affect both animals and humans [2]. The WHO Best Practices for the Naming of New Human Infectious Diseases now discourages the naming of new diseases after geographical locations, people, animals, food, cultural, industry, or occupational references, and terms that incite undue fear, so that the negative impact of disease names can be avoided [5]. Instead, best practices from the WHO dictate that the disease name should contain generic descriptive terms based on disease symptomatology, as well as specific descriptive terms based on how the disease manifests, the target population, and the disease severity or seasonality. The name should likewise reflect the pathogen if it is known [5].

### Impact of a disease nomenclature

Impact of a disease nomenclature The names assigned to diseases can sometimes have unintended negative consequences toward people from the geographic location after which disease was named, as well as toward animals with similar names to a disease. During the COVID‐19 outbreak, some political leaders in the United States [SA2] used terms such as the “China virus” and “Chinese virus” [7] while posting messages to their sizable Twitter following. These terms fueled conspiracy theories and racism [6]. There were reports of increased hate crimes toward Asians, especially in the United States [7]. These events led to social media campaigns with the hashtag “#IAmNotAVirus” [6]. This regrettable situation was clearly what the WHO tried to avoid when they named the disease “COVID‐19” [6], while the scientists at the International Committee on Taxonomy of Viruses named the virus “severe acute respiratory syndrome coronavirus 2 (SARS‐CoV‐2).”

With the incidence of monkeypox rapidly rising, the fear of the virus continues to grow. There have been reports of attacks and killings of monkeys in different cities in Brazil [8]. Brazil presently has over 1700 reported cases and one death [8]. According to the external situational report for the multi‐country outbreak of monkeypox published in August 2022, Brazil has had the highest increase in the number of cases when compared to other countries [3]. Recently, the country reported that at least 10 monkeys were poisoned or attacked, and seven died, in a city in the southeastern part of São Paulo [9]. The National Network to Combat Wild Animal Trafficking (RENCTAS) reported similar attacks in other parts of the country since the emergence of the disease [9].

This is not the first time that monkeys have been targeted in Brazil due to a disease; monkeys were also attacked during yellow fever outbreaks in the past [8]. These attacks occurred during the re‐emergence of yellow fever in Brazil's Southeast and South regions, especially in 2017. The WHO has condemned the attacks aimed at monkeys over fears of the virus spreading in Brazil [8]. The reason for these attacks probably stems from a misunderstanding of the different modes of transmission of the monkeypox virus. One other negative impact that may arise from this “monkeypox” nomenclature is that people who are unaware of the disease may not properly understand the mode of transmission thus putting them at a higher risk of exposure to the disease. Although the monkeypox virus was first detected in monkeys and named after the primates, the name “monkeypox” may be inaccurate because several other animals can be infected, such as rodents, and the natural host of the virus is not currently known [1, 2]. The virus may be transmitted to humans through contact with infected animals [1, 2]. The human‐to‐human transmission occurs by having close physical contact with symptomatic individuals through direct and indirect contact with skin or mucosal lesions of infected persons, body fluids, respiratory droplets, and contaminated objects [1, 2]. This ongoing outbreak has not been linked to animals but instead to human‐to‐human transmissions. Intimate contact during sexual intercourse has been reported as the most frequent mode of transmission (91%) as gathered from the available data [3]. For clarity, this is not a sexually transmitted infection. In other parts of the world excluding Africa, monkeypox tends to primarily affect men, especially men who have sex with men (MSM) due to such intimate physical contact [3]. The WHO has also warned against stigmatizing people who are infected with the virus just because of the large number of cases found in MSM [8].

## CONCLUSION

The WHO is working on alternate names for the monkeypox virus and they are open to suggestions through the ICD proposal platform [3, 10]. Based on the WHO Best Practices for the Naming of New Human Infectious Diseases, the name “monkeypox” is an example of a disease name to be avoided [5]. Some of the recommendations that can be applied to the new name are the use of a short name bearing the causative pathogen with generic descriptive terms [5]. A group that was assembled by the WHO which included virologists and public health experts has agreed on a new name for the clades but there continues to be ongoing work on new names for virus and disease [10]. Henceforth, the former Congo Basin (Central African) and West African clades shall be known as Clade I and Clade II, respectively [10]. Renaming the disease may be a better idea and even though a new name may take time for stakeholders to adjust, it could help clear up the confusion and stigmatization that the “monkeypox” name suggests and perhaps discourage further attacks on these animals both in Brazil and in other parts of the world. It is also of utmost importance to properly educate the public through media campaigns and other means necessary for everyone to understand the signs and symptoms of the virus, as well as the mode of transmission so that people can take precautions to reduce the risk of infection and also stop the spread of misinformation. Moreover, vaccines are available for the post‐exposure prevention of monkeypox in individuals who have had intermediate and high‐risk exposure. People, especially the immunocompromised, should be encouraged to get vaccinated for their safety.

## AUTHOR CONTRIBUTIONS

Lawson Ifeanyi Eya: Conceptualization, Data curation, Formal analysis, Writing – original draft. Muiz Ibrahim: Writing – review & editing. Creuza Rachel Vicente: Supervision, Writing – review & editing. Adrian Paul J. Rabe: Supervision, Final writing – review & editing, Formal analysis. All co‐authors agreed to the final draft of the paper. There was no funding for the development for this paper.

## CONFLICT OF INTEREST

The authors declare no competing interests.

## ETHICS STATEMENT

There is no need for ethical clearance for this type of article.

## Data Availability

None.
